# Editorial

**Published:** 1870-11

**Authors:** 


### Editorial.

EXPLANATION.—The reports of Dr. Jones on Otology, and Dr. Bogue on the Use of Plaster of Paris in Fractures, in the October number of the *Examiner*, should have been credited as read to the Annual Meeting of the Illinois State Medical Society, in May, 1870.

EXCHANGES.—We learn by our exchanges, that a new medical journal has been commenced in Philadelphia, called the *Medical Times;* and that another has been for some time past published at St. Paul, Minnesota. Having never been honored with a copy of either, we cannot give any description of them.

American Medical Association.—We see by our California and Oregon exchanges that the profession in those States is rapidly organizing new medical societies, or reviving old ones, and preparing to give their brethren from this side the mountains a cordial reception at the meeting of the Association next May. And, notwithstanding all the severe criticisms and carping which has been indulged in regarding the last meeting of the Association, we have no doubt but the coming meeting will be one of the most pleasant and profitable that has been held since the Association was organized.

### INFLUENCE OF MUSCULAR EXERCISE ON THE URINE.

Dr. Austin Flint, Jr., read a paper entitled, "The Influence of Excessive and Prolonged Muscular Exercise upon the Elimination of Effete Matters by the Kidneys; based on an Analysis of the Urine passed by Mr. Weston while walking One Hundred Miles in Twenty-one Hours and Thirty-nine Minutes."

When Mr. Edward P. Weston was performing his great pedestrian feat, at the Skating Rink in this city, last May, Dr. Flint visited the rink near the close of the hundred-mile walk. He learned that, by chance, all the urine passed during the walk had been collected in a single vessel. This was placed at his disposal and subjected to a careful quantitative analysis. The observation being entirely unpremeditated, certain elements of scientific completeness were lacking, such as the exact amount of food and drink consumed, etc.; and the specimen of normal urine for comparison was necessarily obtained, not before the walk, but some three months later, when the conditions seemed perfectly satisfactory.

It was evident that an accurate comparison of the urine passed during such an enormous strain upon the muscular and nervous systems, under known conditions of diet, etc., with the normal urine under known conditions, would be of great importance to science; especially since the observations of Fick and Wislicenus, Haughton, and others, had tended to throw doubt upon the commonly received view that exercise increases the elimination of urea.

Mr. Weston is 31 years old; of medium height, rather slight; ordinary weight, without clothing, 122 pounds. From his professional occupation, his hours of sleep and eating are quite regular. He has never taken a course of "training," preparatory to his feats of endurance. His general health is perfect. At the time of the walk he was in "fine" condition, and his weight was five pounds less than ordinary.

The conditions during the walk were, as nearly as could be ascertained, the following:

Weight of body, without clothing, 117 pounds.

Food and drink taken: Beef essence, between one and two bottles; oatmeal gruel, two bottles; sixteen to eighteen eggs, raw, in water; lemonade, half a pint; champagne, about three fluidounces; brandy, two and one-half fluidounces; water to rinse the mouth every few minutes, but little swallowed.

No sleep; no alvine evacuation; perspiration slight.

The urine passed during this time (counted as twenty-two hours) measured 73½ fluidounces (estimated for twenty-four hours at 80 fluidounces); acidity normal; color light canary; odor strongly urinous, but normal; specific gravity, 1011.55; no abnormal matters; microscopical examination negative. Analyzed by the methods detailed in the speaker's manual of the "Chemical Examination of the Urine," its composition per fluidounce was found to be:

*Urine of Exercise per Fluidounce.*

| | |
|---|---|
| Urea | 5.779 grs. |
| Chlorine | 1.120 " |
| Sulphuric acid | 0.920 " |
| Phosphoric acid (total) | 1.504 " |
| Phosphoric acid (with alkalies) | 1.280 " |
| Phosphoric acid (with earth) | 0.224 " |
| Uric acid | 0.500 " |

The conditions during the twenty-four hours, 6 P.M. to 6 P.M., selected for collecting the standard specimen of urine for comparison, were as follows:

Weight, naked, 122 pounds.

Food and drink: Supper—Pickled lamb's tongues, warm light biscuit, one cup tea. Dinner—Cold corned-beef, hot bread-cakes, one slice bread, one cup coffee. One glass of effervescing root beer, and two cigars during day. Had eaten salt ham three hours before the beginning of the selected twenty-four hours.

Sleep between eleven and twelve hours; one alvine discharge (no urine lost).

The urine passed in the twenty-four hours measured 33 fluidounces; acidity rather faint; color light canary, slightly turbid; odor strongly urinous, but normal; specific gravity 1025.43; no abnormal matters; decomposition rather rapid; microscopical examination showed slight excess of mucus, otherwise negative. Analyzed by methods identical with the last, the composition per fluidounce was found to be:

*Urine of Rest per Fluidounce.*

| | |
|---|---|
| Urea | 5.800 grs. |
| Chlorine | 3.360 " |
| Sulphuric acid | 1.440 " |
| Phosphoric acid (total) | 0.960 " |
| Phosphoric acid (with alkalies) | 0.640 " |
| Phosphoric acid (with earths) | 0.320 " |
| Uric acid | 0.680 " |

The Urine of Exercise, calculated for twenty-four hours, is compared with the Urine of Rest for twenty-four hours in the following table:

*Urine of Rest and of Exercise for Twenty-four Hours.*

| | Rest. | Exercise. |
|---|---|---|
| Total quantity | 33.000 fl. oz. | 80.000 fl. oz. |
| Urea | 191.400 grs. | 463.368 grs. |
| Chlorine | 110.880 " | 89.808 " |
| Sulphuric acid | 47.520 " | 73.776 " |
| Phosphoric acid (total) | 31.680 " | 120.600 " |
| Phosphoric acid (with alkalies) | 21.120 " | 102.600 " |
| Phosphoric acid (with earths) | 10.560 " | 18.000 " |
| Uric acid | 22.440 " | 40.080 " |

After making all allowances for differences of diet and other conditions, the speaker concluded that the severe and prolonged muscular exertion must be credited with producing a decided increase in the amount of water eliminated by the kidneys; an increase of from seventy-five to one hundred per cent. in the amount of urea eliminated; a considerable part of the increase in the sulphates and phosphates; and an increase of about seventy-eight per cent. in the uric acid. The dimunition of the chlorides seemed pretty well accounted for by the diet.

For further calculations from the above data, and a more detailed statement of the points involved, we refer the reader to Dr. Flint's paper, which is to be published.*

The Society adjourned.—*N. Y. Medical Record.*

*It appears in the *New York Medical Journal* for October.—Ed.

### POISONING BY CARBOLIC ACID, IN A CASE OF ACUTE ECZEMA.

Reported by T. G. RODDICK, M.D., Assistant House Surgeon, Montreal General Hospital.

Thomas Hobbs, æt. 80, was admitted into the Montreal General Hospital, under care of Dr. Fraser, on the 18th April, 1870, suffering from Acute Eczema, intense in degree, and affecting the whole cutaneous surface. The patient had been troubled with the disease for about five weeks previous to admission, and had been treated for scabies with the ordinary sulphur ointment. The arms, legs, and trunk were literally covered with the disease, and it had invaded his scalp for a short distance behind. He was very feeble, and indeed had to be assisted in and out of bed.

For the first three or four days after admission, he was ordered ungi. zinzi., to be applied over the diseased surface, twice a-day, and in the interim a tepid bath. This plan of treatment had no marked effect on the disease, so on the fifth day the dresser was instructed to apply on lint an ointment containing one part of carbolic acid to four parts of lard, over the arms and thighs, and to cover the whole with oil silk. This application was faithfully made about four o'clock in the afternoon, and at half-past five the nurse reported that the old man was dying. When seen, as he was almost instantly, he was found to be in profound coma, with the pupils firmly contracted; breathing stertorous; pulse weak, quick, and flickering; whole surface of the body livid; extremities cold; large quantity of mucus in bronchial tubes; inability to swallow; profound insensibility. The patients in the same ward had seen him, half an hour before, crawl out of bed, and, after sitting on the chair a few moments, fall to the floor apparently in a faint. He was lifted to his bed and taken no further notice of till the nurse gave the alarm.

It was thought that the extensive application of carbolic acid would account for his condition. So, accordingly, the dressings were instantly thrown off and the part washed thoroughly with soap and water. At the same time sinapisms were applied to his chest and the calves of his legs, and a blister to the nape of his neck; brandy was given as freely as possible, and a turpentine and castor oil enema. For the first hour his condition improved rapidly, but as soon as the stimulating effect of the brandy and sinapisms had passed off, he seemed to lapse into his former condition. The symptoms varied in intensity from time to time, until ten o'clock, when he vomited freely, and from that time rapidly regained his consciousness and fell almost immediately into a natural sleep.

The odor of carbolic acid in the vomited matter was distinctly perceptible, but, unfortunately, none of the secretions were tested.

When fully recovered, the patient said that a very few minutes after the application of the ointment he experienced a peculiar burning, prickling sensation over the whole body, and that although he had the greatest desire to micturate, he could not pass a single drop of urine. He had no recollection of getting out of bed, and that he was in a faint when the patients found him on the floor there can be little doubt.

As to the disease it improved with marvellous rapidity, and although nothing was afterwards applied but cod liver oil, he was pronounced cured on the sixteenth day after admission, and has had no recurrence of the disease since.

Dr. Fraser, in a few remarks to the students, stated that the case is instructive in two respects: 1st. As regards the danger incurred by the extensive application of carbolic acid to the skin, when the cuticle is removed, as it always is in eczema, leaving the cutaneous absorbents and capillaries exposed, through which it is readily absorbed and produces its known depressing effects upon the circulation through the nervous system. These effects have also been occasionally observed to follow its injection into large abscesses. 2d. As regards its efficiency as a therapeutic remedy in eczema, in which disease, however, judging from its effects in the present case, it should be employed with caution, or to portions only of the diseased surface at a time, and its effects closely watched.

The action of the acid on the urinary organs which has been observed by others, was also pointed out, and so was the treatment which succeeded so well in combating the dangerous effects in the present instance.—*Canada Medical Journal.*

---

The Photographic Review of Medicine and Surgery, a publication now, for the first time offered to the profession, has for its object the presentation of the most interesting cases coming under notice. The *Photographic Review* will appear in numbers, every other month, each containing four photographic plates, with appropriate notes and remarks. Subscription, $6.00 per annum. J. P. Lippincott & Co., publishers, Philadelphia.—*Boston Medical and Surgical Journal.*

### Mortality for the Month of September, 1870.

| | |
|---|---|
| Accidents, by fire, Farwell building | 5 |
| " burned, clothes taking fire | 1 |
| " blow from bat | 1 |
| " drowned | 8 |
| " crushed by fall of stone | 1 |
| " by fall | 3 |
| " fracture of skull | 2 |
| " thrown from wagon | 1 |
| " run over by " | 1 |
| " railroad | 5 |
| " shot | 1 |
| " scalded | 1 |
| Abscesses scrofulous | 1 |
| " pulmonic | 1 |
| Apoplexy | 5 |
| Ascites | 1 |
| Bowels, congestion of | 1 |
| " obstruction of | 1 |
| Brain, congestion | 6 |
| " inflammation | 8 |
| Births, premature | 27 |
| Births still | 49 |
| Bronchitis | 4 |
| " capillary | 1 |
| " chronic | 1 |
| Cancer of breast | 2 |
| " stomach | 1 |
| " uterus | 2 |
| " neck | 1 |
| Carbuncle of head and back | 1 |
| Childbirth | 1 |
| Cholera Infantum | 99 |
| " morbus | 1 |
| Chest, gun-shot wound of long standing | 1 |
| Consumption | 35 |
| Convulsions | 65 |
| Croup | 21 |
| " membranous | 10 |
| Cynanche tonsillaris | 2 |
| Cysterarcoma & Dropsy | 1 |
| Development deficient | 1 |

| | |
|---|---|
| Debility, general | 9 |
| Diarrhœa | 29 |
| " chronic | 14 |
| " & complications | 4 |
| Diphtheria | 20 |
| Dropsy, general | 5 |
| " of chest | 1 |
| Dyspepsia | 1 |
| Dysentery | 32 |
| " acute | 1 |
| " chronic | 2 |
| " typhoid | 3 |
| Entero-colitis | 4 |
| Enteritis | 11 |
| Erysipelas | 1 |
| " of leg | 1 |
| Fever, congestive | 2 |
| " puerperal, | 2 |
| " intermittent | 1 |
| " scarlet | 5 |
| " " complications | 2 |
| " " malignant | 1 |
| " typhoid | 58 |
| " typhus | 1 |
| Gastritis | 3 |
| Gastro-Enteritis | 4 |
| Hæmatemesis | 1 |
| Hemorrhage umbilical | 1 |
| " internal | 1 |
| Heart, disease of | 1 |
| " mitral valve, insufficiency of | 1 |
| " organic disease | 2 |
| " fatty degenerat'n | 1 |
| " valvular disease | 1 |
| Hemiplegia | 1 |
| Hepatitis, | 1 |
| Hernia, injuinal | 1 |
| " strangulated | 1 |
| Hip-disease | 1 |
| Hydrocephalus | 7 |
| " acute | 4 |
| " chronic | 1 |
| Inanition | 13 |
| Imflam. from biliary calculi | 1 |

| | |
|---|---|
| Intemperance | 5 |
| Ischuria, renal | 1 |
| Kidneys, Bright's disease | 1 |
| Liver, disease & dropsy | 1 |
| " cirrhosis | 1 |
| " hæmorrhage | 1 |
| " Induration | 1 |
| Lupus, exedens | 1 |
| Lungs, congestion | 5 |
| " " and old age | 1 |
| " hæmorrhage | 1 |
| Mania, acute | 1 |
| Manslaughter | 2 |
| Metro peritonits puerperal | 1 |
| Meningitis | 7 |
| " cerebro-spinal | 2 |
| " tubercular | 4 |
| Neck, tumor on | 1 |
| Old age | 5 |
| " congestion lungs | 1 |
| " and dysentery | 1 |
| Paralysis | 1 |
| Pericarditis | 1 |
| Peritonitis & Ascitis | 1 |
| Phlegmasia dolens | 1 |
| Pharngitis & Bronchitis | 1 |
| Pneumonia | 11 |
| " bronche | 1 |
| " typhoid | 5 |
| Scrofula | 1 |
| Septiceamia | 1 |
| Spina bifida | 1 |
| Stomatitis | 1 |
| Suicide by arsenic | 2 |
| Syphilis, secondary | 1 |
| Tabes mesenterica | 33 |
| Teething | 9 |
| " & complications | 8 |
| Tumor ovarian | 1 |
| Uterus, fibroid tumors | 1 |
| " hæmorrhage | 1 |
| Whooping-cough | 7 |
| Total | 691 |

### COMPARISON.

Deaths in Sept., 1870, 691 | Deaths in Sept., 1869, 814 | Decrease, 123
Deaths in Aug., 1870, 1037 | Decrease, 346

### AGES.

| | | | | | |
|---|---|---|---|---|---|
| Under 1 | 229 | 10 to 20 | 36 | 70 to 80 | 16 |
| 1 to 2 | 143 | 20 to 30 | 65 | 80 to 90 | 3 |
| 2 to 3 | 34 | 30 to 40 | 41 | Unknown, | 1 |
| 3 to 4 | 12 | 40 to 50 | 39 | | |
| 4 to 5 | 9 | 50 to 60 | 24 | Total, | 691 |
| 5 to 10 | 15 | 60 to 70 | 24 | | |

| | | | | | |
|---|---|---|---|---|---|
| Males, | 389 | Females, | 302 | Total, | 691 |
| Single, | 534 | Married | 157 | Total, | 691 |
| White, | 684 | Colored, | 7 | Total, | 691 |

NATIVITY.

| | | | | | |
|---|---|---|---|---|---|
| Atlantic Ocean | 0 | England | 14 | Nova Scotia | 0 |
| Bohemia | 8 | France | 1 | Switzerland | 1 |
| Canada | 4 | Germany | 81 | Scotland, | 0 |
| Chicago, Native | 79 | Holland | 0 | Sweden | 20 |
| Chicago, Foreign | 329 | Ireland | 60 | Unknown | 6 |
| U. S., other parts | 79 | Isle of Jersey | 0 | | |
| Denmark | 1 | Norway | 8 | Total, | 691 |

MORTALITY BY WARDS FOR THE MONTH.

| Wards. | Mortality. | Wards. | Mortality. |
|---|---|---|---|
| 1 | 5 | 18 | 53 |
| 2 | 13 | 19 | 14 |
| 3 | 21 | 20 | 12 |
| 4 | 13 | Accidents | 30 |
| 5 | 18 | County Hospital | 18 |
| 6 | 57 | Home for Friendless | 2 |
| 7 | 44 | Hospital Alexian Brothers | 1 |
| 8 | 64 | Immigrants | 13 |
| 9 | 49 | Jewish Hospital | 1 |
| 10 | 14 | Mercy Hospital | 3 |
| 11 | 20 | Marine Hospital | 2 |
| 12 | 32 | Manslaughter | 2 |
| 13 | 11 | St. Joseph Orphan Asylum | 6 |
| 14 | 12 | Suicide | 2 |
| 15 | 75 | | |
| 16 | 40 | Total, | 691 |
| 17 | 44 | | |

---

Secretion of Milk Restored by Electricity.—A married lady had had two children, and in each case the flow of milk was abundant. Had typhoid fever in August, 1869. Confined Jan. 8th, 1870, for the third time. Milk appeared in the breasts on the third day, but on the fourth it diminished, and for several days it was almost wholly absent. On the evening of the 13th, the interrupted current from a Farmer's battery was applied, for about $2\frac{1}{2}$ minutes, to one side of the breast, and then, for the same length of time, to the other side, the poles being covered with moistened sponge. Little pain and but slight redness accompanied the operation. The milk had increased very decidedly on the next morning, and after five days' use of the current, with a daily application, it was quite abundant. Reference was made to Dr. Barker's paper on this subject in the *New York Medical Journal.—Boston Medical and Surgical Journal.*

Receipts from September 1, to October 25.—Dr. H. C. Hutchinson, City, $3.00; Dr. T. J. Whitter, Irving, Ill., 3.00; Dr. Milton Parker, City, 6.00; Dr. L. D. Tompkins, Cassopolis, Ill., 3.00; Dr. Thos. Aiton, Pittsfield, Ill., 3.00; Dr. Curtis, City, 3.00; Dr. A. L. Shay, Ossion, Iowa, 3.00; Dr. S. A. McWilliams, 3.00; Dr. T. W. Havres, Mt. Hope, Wis., 3.00; Dr. J. F. Hopkins, Oconomowoc, Wis., 6.00; Dr. J. Schenck, City, 3.00; Dr. R. O. Phillips, City, 3.75; Dr. J. R. Garrell, Newton, 9.00; Dr. W. R. Fox, San Leardo, Col., 3.00; Dr. G. W. Rohr, Rockford, Ill., 6.00; Dr. O. T. Bundy, Deposit, N. Y., 3.00; Dr. J. W. Johns, Dyer, Ind., 5.00.

